# Synthesis and Properties of Side-Chain Functionalized Polytetrahydrofuran Derivatives via the Blue-Light Photocatalytic Thiol-Ene Reaction

**DOI:** 10.3390/polym11040583

**Published:** 2019-04-01

**Authors:** Xiuzhong Zhu, Ting Bai, Zichao Wang, Jie Liu, Xin Min, Tong Wang, Wanbin Zhang, Xiaodong Fan

**Affiliations:** 1 The Key Laboratory of Space Applied Physics and Chemistry, Ministry of Education and Shaanxi Key Laboratory of Macromolecular Science and Technology, School of Science, Northwestern Polytechnical University, Xi’an 710072, China; zhuxiuzhong321@163.com (X.Z.); baiting_zoe@mail.nwpu.edu.cn (T.B.); wc19910102@126.com (Z.W.); liujie509_1982@126.com (J.L.); 15991672082@163.com (X.M.); 17795837806@163.com (T.W.); 2 Shaanxi Collaborative Innovation Center of Industrial Auxiliary Chemistry and Technology, Shaanxi University of Science and Technology, Xi’an 710021, China; zhangwanbin@sust.edu.cn

**Keywords:** unsaturated polytetrahydrofuran, blue-light photocatalytic thiol-ene reaction, side-chain functionalized polytetrahydrofuran derivatives, backbone-thermoresponsive

## Abstract

A series of side-chain functionalized polytetrahydrofuran (PTHF) derivatives were synthesized via the blue-light photocatalytic thiol-ene “click” reaction. Firstly, unsaturated polytetrahydrofuran (UPTHF) as a new unsaturated polyether was synthesized via condensation polymerization of cis-2-butene-1,4-diol and trans-1,4-dibromo-2-butene using potassium hydroxide (KOH) as a catalyst. Then, double bonds in the backbone of UPTHF were modified into different pendant functionality side groups by blue-light photocatalytic thiol-ene “click” reaction using Ru(bpy)_3_Cl_2_ as a photoredox catalyst, obtaining different side-chain functionalized PTHF derivatives. The structure and the morphology of the side-chain functionalized PTHF derivatives was characterized via Fourier-transform infrared spectra (FTIR), nuclear magnetic resonance (NMR), size exclusion chromatography/multi-angle laser light scattering (SEC/MALLS), and differential scanning calorimeter (DSC). The results showed that the blue-light photocatalytic thiol-ene reaction exhibited high efficiency, and all the unsaturated bonds were modified. Different branch units bestowed different performance of PTHF derivatives; we systematically investigated the thermal properties, pH-triggered and temperature-triggered, self-assembly behaviors of different PTHF derivatives.

## 1. Introduction

Polytetrahydrofuran (PTHF) is an extremely versatile polyether used as adhesives and biomedical and elastomeric materials [1,2,3,4,5]. As a commodity telechelic polymer, ~200,000 tons of PTHF are produced annually [6]. For the foreseeable future, the PTHF derivatives should also prove to be useful in a wide range of applications. However, a fundamental challenge with all PTHF-based systems is the lack of functional handles along the polymer backbone, which limits the modification and tunability of this valuable material’s platform, so that the preparation of the PTHF derivatives just are a,ω-telechelic poly(THF)s [7,8]. Therefore, the synthesis of the side-chain functionalized PTHF derivatives with a precisely defined molecular weight, composition, and architecture can still pose a significant challenge, and have not been reported in the literature yet. 

As is known, post-polymerization modification is a very effective strategy for synthesis of functionalized polymers [9,10,11,12]. For instance, functionalized polyethylene has been obtained through functionalization reactions of polybutadiene (PB) [13]. This is because the internal bond of PB can be reacted with a range of functional molecules. This method successfully solved the limitation that the common industrial techniques had no way to synthesize functionalized polyethylene. Inspired by this, we designed a new polyether named unsaturated polytetrahydrofuran (UPTHF), and then through post-modification of UPTHF got a chance to obtain side-chain functionalized PTHFs [14,15]. Among the various ways about the post-modification of unsaturated polymers, the most widely used is the thiol-ene “click” reaction.

Recently, the thiol-ene “click” reaction has been widely applied in organic and polymer syntheses as well as the preparation of gels, thermosets, and the post-modification of polymers [16,17,18,19,20]. In general, thermal or UV light is required to induce thiol-ene “click” reaction by using corresponding radical initiators; among the several available methods, the technique employing the UV light was the most widely used [21,22,23]. For instance, Schlaad and co-workers successfully prepared polyolefin derivatives via the thiol-ene “click” reaction by ultraviolet (UV) irradiation and adopting polybutadiene with a high content of 1,2-units as a polymer precursor; they also investigated the photoinitiation polymerization mechanism in detail [24]. As one of the available living polymerization methods, the ring-opening metathesis polymerization (ROMP) is a versatile and an efficient synthetic strategy for the polymerization of cyclic olefins by using metal alkylidene initiators, which create a series of polyolefin polymers. Simultaneously, UV light photocatalytic thiol-ene reaction is also widely used to modify those polyolefin polymers [25,26]. Hence, we initially intended to synthesize side-chain functionalized PTHFs via the UV light photocatalytic thiol-ene reaction, but unfortunately, the double bonds in the UPTHF backbone could not be fully transformed; indeed, only a part of double bonds was involved in the reaction, when the reaction time was set limited to 24 h. The underlying cause creating this issue was likely that in-chain double bonds of UPTHF exhibited a low-activity, which was in agreement with the experimental observation that in-chain double bonds of polybutadiene were 10 times less reactive than vinyl double bonds [27]. Moreover, the use of the UV light implies the development of harsh and expensive conditions for the functionalization of polymers. Recently, it was reported that thiol-ene reaction could be initiated at room temperature by visible light using novel visible-light-absorbing transition metal photocatalysts, becoming widely available for the synthesis of small molecular compounds [28,29,30,31]. As for polymers, Boyer and Zhang reported the thiol-ene modification of polybutadiene using visible light, showing that all double bonds were converted in a short time [32,33].

**Figure 1:**
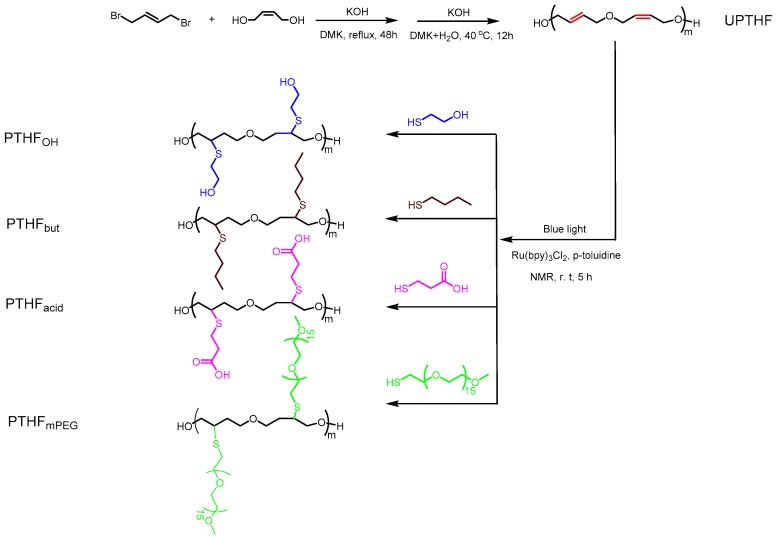
The synthesis routes of unsaturated polytetrahydrofuran (UPTHF) and side-chain functional polytetrahydrofuran (PTHF) derivatives.

Based on the above considerations and inspired by previous research outcomes, we reported a simple strategy to synthesize UPTHF via condensation polymerization and then post-polymerization modification of UPTHF to obtain side-chain functionalized PTHFs via the blue-light photocatalytic thiol-ene reaction. UPTHF was synthesized via the condensation polymerization using cis-2-butene-1,4-diol and trans-1,4-dibromo-2-butene as monomers. Then, blue light was used to induce the thiol-ene reaction between UPTHF and a range of thiol-containing compounds, such as mercaptoethanol, n-butyl mercaptan, mercaptopropionic acid, as well as methoxy- polyethylene glycol-thiol (mPEG-SH) (*M*_n_ = 750 g mol^−1^), to obtain different PTHFs named PTHF_OH_, PTHF_but_, PTHF_acid_, and PTHF_mPEG_, respectively Figure 1). Afterward, we characterized the structure and the morphology of the polymers via FTIR, ^1^H NMR, ^13^C NMR, size exclusion chromatography/multi-angle laser light scattering (SEC/MALLS), and differential scanning calorimeter (DSC). The pH-triggered self-assemble behaviors of PTHF_acid_ and temperature-triggered self-assemble behaviors of PTHF_mPEG_ were investigated via various methods, including dynamic light scattering DLS, and UV-Vis spectroscopy. The biocompatibility of PTHF_acid_ and PTHF_mPEG_ was also studied via the in vitro cytotoxicity assay [34,35]. The overall experimental procedure is illustrated in Figure 1.

## 2. Experimental Section

### 2.1. Materials

Cis-2-butene-1,4-diol (97%, Macklin, Shanghai, China), trans-1,4-dibromo-2-butene (98%, Alfa Aesar, Heysham, UK), tris(acetonitrile)cyclopentadienylruthenium(II) hexafluorophosphate ([CpRu(CH_3_CN)_3_]PF_6_, 98%, Acros, BE), Quinaldic acid (98%, Aladdin, Shanghai, China), Ru(bpy)_3_Cl_2_ (98%, Adamas, Basel, Switzerland), and *p*-toluidine (98%, Adamas, Basel, Switzerland) were used as received, and 2-mercaptoethanol, n-butyl mercaptan and 3-mercaptopropionic acid were purchased from Macklin. mPEG_16_-SH was bought from Shanghai ToYong Bio Company. Terahydrofuran (THF, 99.9%, Alfa Aesar, Heysham, UK), dichloromethane (DCM, 99.5%, Sinopharm Chemical Reagent Co., Ltd., Tianjin, China), diethyl ether (99.5%, Alfa Aesar, Heysham, UK), and acetone (9.5%, Sinopharm Chemical Reagent Co., Ltd., Tianjin, China) were refluxed over sodium wire (Na, 98.0%, Sinopharm Chemical Reagent Co., Ltd., Tianjin, China) in the presence of benzophenone until a blue color presence, and was distilled before use. Methanol, *N*-methyl-2-pyrrolidone (NMP), and other organic solvents were all purchased from Sinopharm Chemical Reagent Co., Ltd. (SCRC, Tianjin, China) and used without any purification. 

### 2.2. Synthesis of Unsaturated Polytetrahydrofuran (UPTHF)

A 50 mL flask loaded with a stir bar, cis-2-butene-1,4-diol (880 mg, 10 mmol), trans-1,4-dibromo-2-butene (2139 mg, 10 mmol) and potassium hydroxide (KOH, 1685 mg, 30 mol) were dissolved in solution of acetone (15 mL). The solution was stirred with a reflux condenser at 65 °C for 72 h, and then 2 mL purified water was added into the flask, keeping reaction for 24 h at 40 °C. The polymer was extracted using DCM, and then was precipitated into an excess of cold n-hexane. The obtained brown viscous liquid was redissolved with 2 mL of THF and precipitated again with methanol. The polymer was washed three times with methanol and dissolved in 5 mL dichloromethane. Finally, the polymer was obtained by rotary evaporation removal of DCM. The final yield of UPTHF was 81% (1280 mg). *M*_n,SEC_ = 4300 g mol^−1^, *M*_w_/*M*_n_ = 1.48. ^1^H NMR (DMSO-*d*_6_, *δ*): 5.74 (–C***H***=C***H***–, trans-1,4), 5.62 (–C***H***=C***H***–, cis-1,4), 4.7 (–C***H***_2_–OH), 3.99 (–C***H***_2_–C***H***_2_–, cis-1,4), 3.92 (–C***H***_2_–C***H***_2_–, trans-1,4). 

### 2.3. Synthesis of Side-Chain Functionalized PTHFs 

#### 2.3.1. Synthesis of PTHFs with a Hydroxy Branching Unit (PTHF_OH_)

PTHF derivative with a hydroxy branching unit (PTHF_OH_) was synthesized by blue-light photocatalytic thiol-ene reaction between UPTHF and mercaptoethanol. UPTHF (260 mg, –C=C–, 3.7 mmol), mercaptoethanol (3.16 mL, 45 mmol), Ru(bpy)_3_Cl_2_ (4.5 mg, 7 × 10^−3^ mmol), and *p*-toluidine (30 mg, 0.28 mmol) were dissolved in NMP and irradiated by a household blue LED lights bulb (7 W) for 5 h at room temperature. After reaction, unreacted mercaptoethanol and NMR were isolated by dialyzed (molecular weight cut off: 300) against distilled water for 48 h, and the final product was obtained by freeze drying. ^1^H NMR (DMSO-*d*_6_, *δ*): 4.75 (–O***H***), 3.87–3.18 (–C***H***_2_–O–), 2.90 (–(CH_2_)_2_–C***H***–S), 2.62 (–C***H***_2_–S–), 1.92–1.55 (–CH_2_–C***H***_2_–).

#### 2.3.2. Synthesis of PTHFs with a Carboxyl Branching Unit (PTHF_but_)

PTHF derivative with a butyl branching unit (PTHF_but_) was synthesized by blue-light photocatalytic thiol-ene reaction between UPTHF and n-butyl mercaptan. UPTHF (260 mg, –C=C–, 3.7 mmol), n-butyl mercaptan (4.83 mL, 45 mmol), Ru(bpy)_3_Cl_2_ (4.5 mg, 7 × 10^−3^ mmol) and *p*-toluidine (30 mg, 0.28 mmol) were dissolved in NMP and irradiated by a household blue LED lights bulb (7 W) for 5 h at room temperature. After reaction, unreacted n-butyl mercaptan and NMR were isolated by dialyzed (molecular weight cut off: 300) against mix solution of methyl alcohol and distilled water for 48 h, and the final product was obtained by rotary evaporation removal of methyl alcohol and then freeze dried. ^1^H NMR (DMSO-*d*_6_, *δ*): 3.67–3.17 (–C***H***_2_–O–), 2.98–2.37 (–C***H***_2_–S–C***H***–), 2.01–1.25 (CH_3_–C***H***_2_–C***H***_2_–, –C***H***_2_–O–), 0.87 (C***H***_3_–CH_2_–).

#### 2.3.3. Synthesis of PTHF Derivative with a Carboxyl Branching Unit (PTHF_acid_)

PTHF derivative with a carboxyl branching unit (PTHF_acid_) was synthesized by blue-light photocatalytic thiol-ene reaction between UPTHF and 3-mercaptopropionic acid. UPTHF (260 mg, –C=C–, 3.7 mmol), 3-mercaptopropionic acid (3.95 mL, 45 mmol), Ru(bpy)_3_Cl_2_ (4.5 mg, 7 × 10^−3^ mmol) and *p*-toluidine (30 mg, 0.28 mmol) were dissolved in NMP and irradiated by a household blue LED lights bulb (7 W) for 5 h at room temperature. After reaction, unreacted 3-mercaptopropionic and NMR were isolated by dialyzed (molecular weight cut off: 300) against distilled water for 48 h, and the final product was obtained by freeze drying. ^1^H NMR (DMSO-*d*_6_, *δ*): 12.20 (–COO***H***), 2.49 (–C***H***_2_–COOH), 2.66 (–C***H***_2_–S–), 1.50 (saturation protons in –C***H***_2_–).

#### 2.3.4. Synthesis of PTHF Derivative with a mPEG Branching Unit (PTHF_mPEG_)

A series of PTHF_mPEG_ with different degrees of functionalization were synthesized by blue-light photocatalytic thiol-ene reaction between UPTHF and mPEG-SH. UPTHF (33 mg, 0.01 mmol), mPEG-SH (75 mg, 10 mmol; 150 mg, 20mmol, 750 mg, 100mmol), Ru(bpy)_3_Cl_2_ (2.25 mg, 3.5 × 10^−3^ mmol) and *p*-toluidine (15 mg, 0.14 mmol) were dissolved in NMP and irradiated by a household blue LED lights bulb (7 W) for a certain time at room temperature. After reaction, unreacted mPEG-SH and NMR were isolated by dialyzed (molecular weight cut off: 1000) against distilled water for 48 h, and the final product was obtained by freeze drying. ^1^H NMR (DMSO-*d*_6_, *δ*): 3.64 (–C***H*_2_**–CH_2_–S–), 3.52 (–C***H***_2_–C***H***_2_–OH), 3.43 (–C***H***_2_–O–), 2.90 (–C***H***_2_–S–), 2.69 (–C***H***–S–), 2.01 (–C***H***_2_–CH_2_–O–).

### 2.4. Measurements

Fourier-transform infrared spectra (FTIR) were obtained on a Nicolet iS10 FT-IR instrument (Nicolet Instrument Corporation, Madison, WI, USA) in the region from 4000 to 500 cm^−1^. The ^1^H NMR and ^13^C NMR spectra were carried out on a Bruker 400 MHz NMR spectrometer (Bruker Corporation, Karlsruhe, Germany) using deuterated chloroform (CDCl_3_) or deuterated dimethylsulfoxide (DMSO-*d*_6_) as solvents, and tetramethylsilane (TMS) as the internal standard. The molecular weights and polydispersity indexes of the polymers were determined on a Wyatt DAWN EOS SEC-MALLS, THF was used as the eluent with a flow rate of 0.5 mL min^−1^ at 25 °C.

The glass transition and melting behaviors of polymers were investigated via DSC using a DSC200PC thermal analysis system (Netzsch Instruments, Selb, Germany). Samples weighing approximately 8 mg were scanned at a rate of 10 °C min^−1^ from −90 to 100 °C. To remove thermal history, all the samples were first heated to 125 °C and, subsequently, cooled down to −90 °C; the samples were held at this temperature for 5 min before the scan.

The optical transmittance of polymer solutions at varying pH or temperature values were measured by UV-Vis spectroscopy (Shimadzu UV-2550, Kyoto, Japan) at a wavelength of 500 nm.

The hydrodynamic diameter, diameter distribution, as well as zeta potentials of the aggregates were determined by dynamic light scattering (DLS, Malvern Instruments, Malvern, UK) at different pH or temperature values. The scattered light of a He-Ne laser at 633 nm was measured at an angle of 173°. All the samples were measured directly without any filtration.

The in vitro cytotoxicity of the PTHF_mPEG2_ and the viability of A549 cells were evaluated by using the cell counting kit (CCK-8) assay. The A549 cells in dulbecco’s modified eagle medium (DMEM) supplemented with 10% fetal bovine serum (FBS) were seeded into 96-well plates at a density of 1 × 10^4^ cells per well and cultured for 24 h at 37 °C under CO_2_/air (5/95, *v*/*v*). Then, the cells were cultured with a medium containing various concentrations of PTHF_mPEG2_ from 50 to 750 μg mL^−1^. After the cells were incubated for 24 h, 10 μL of CCK-8 solution was added to each well and the cells were incubated for another 2 h at 37 °C. Cell viability was determined by using a microplate reader of absorbance at 450 nm.

## 3. Results and Discussion

### 3.1. Synthesis of UPTHF

**Figure 2:**
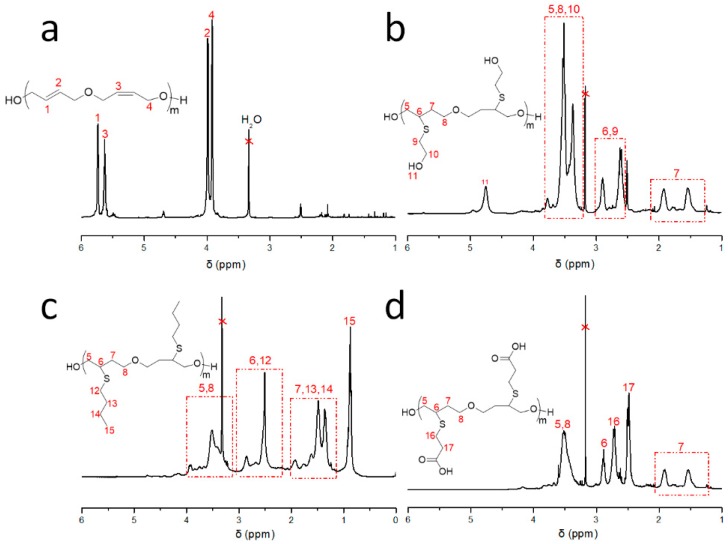
^1^H NMR spectra of UPTHF ((**a**), Sample 2 in Table 1), PTHF_OH_ (**b**), PTHF_but_ (**c**) and PTHF_acid_ (**d**) in DMSO-*d*_6_.

In order to obtain a high molecular weight of UPTHF, polycondensations of UPTHF were performed using cis-2-butene-1,4-diol and trans-1,4-dibromo-2-butene as a monomer at different process conditions, resulting in a series of polymers with number-average molecular weight (*M*_n_) ranging from 279 to 4300 g mol^−1^ and a dispersity index of ~1.5 (Table 1). As shown in Table 1, the *M*_n_ of UPTHF was only 279 g mol^−1^, if potassium carbonate (K_2_CO_3_) was used as a catalyst. Meanwhile, the *M*_n_ of the UPTHF was 423 g mol^−1^ using sodium hydride (NaH) as a catalyst (Table 1, Sample 1). Experimental results have shown: By using KOH as a catalyst, the *M*_n_ of UPTHF was 4306 g mol^−1^ reaction for 72 h (Table 1, Sample 2). Therefore, KOH is an appropriate catalyst for the synthesis of UPTHF. The ^1^H NMR spectrum for UPTHF (*M*_n_ = 4300 g mol^−1^) is provided in Figure 2a. The integral ratio of the peaks at δ = 5.74 and 5.63 ppm, which are attributable to the double bone protons resonance in trans-2-butenyl ether and cis-2-butenyl ether, is 1.00:0.95, which is very close to 1:1, indicating that UPTHF has been successfully synthesized (Figure 2a and Figure S1).

### 3.2. Synthesis of the Side-Chain Functionalized PTHFs

**Figure 3:**
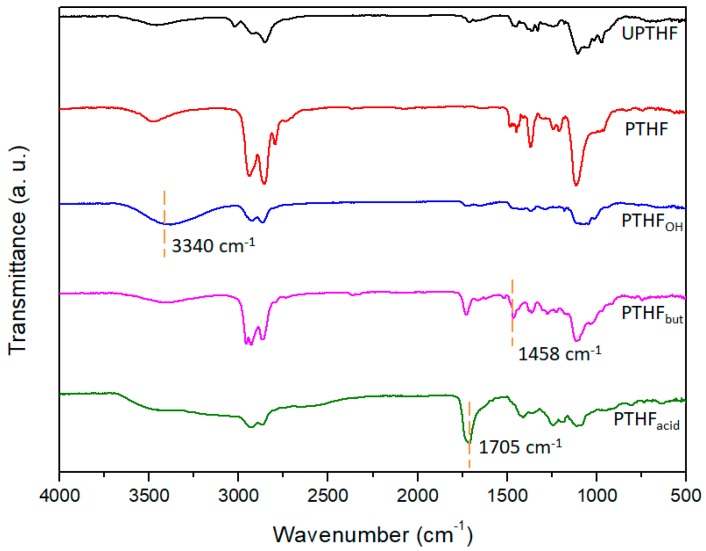
FTIR spectra of UPTHF, PTHF, PTHF_OH_, PTHF_but_ and PTHF_acid_.

**Figure 4:**
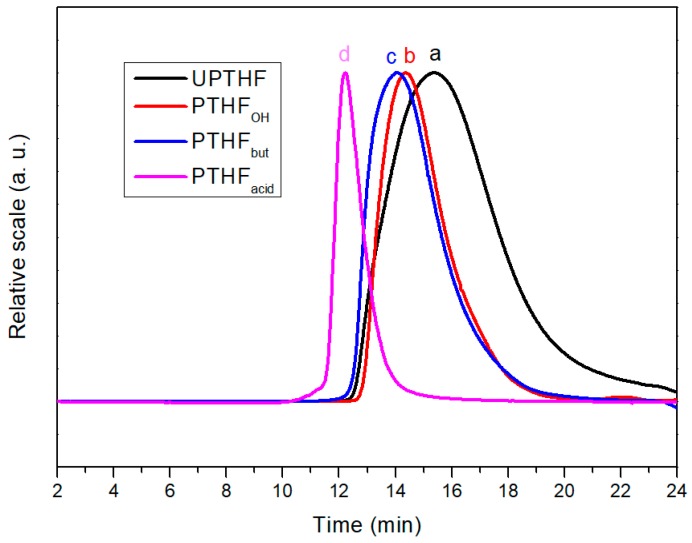
Size exclusion chromatography (SEC) elution curves of UPTHF ((**a**), sample 2 in Table 1), PTHF_OH_ (**b**), PTHF_but_ (**c**) and PTHF_acid_ (**d**). Terahydrofuran (THF) was used as the eluent.

The preparation of the side-chain functionalized PTHFs, such as PTHF_OH_, PTHF_but_, and PTHF_acid_ was performed in similar process conditions. Blue-light (7 W) photocatalytic thiol-ene reaction of UPTHF (260 mg, *M*_n_ = 4300 g mol^−1^, 0.079 mmol, –C=C–, 3.7 mmol) and mercaptoethanol, n-butyl mercaptan, or mercaptopropionic acid (45 mmol) in the presence of Ru(bpy)_3_Cl_2_ (2.25 mg, 3.5 × 10^−3^ mmol) and *p*-toluidine (5.0 mg, 0.05 mmol) in *N*-methyl pyrrolidone (NMP, 3 mL) led to the formation of PTHF_OH_, PTHF_but_, and PTHF_acid_, respectively. The ^1^H NMR spectra of PTHF_OH_, PTHF_but_, and PTHF_acid_ are displayed in Figure 2b–d, respectively. It can be seen that the peaks at δ = 5.74 and 5.63 ppm (associated to the double bond protons resonance in UPTHF, shown in Figure 2a) have all disappeared, whereas three new peaks appear: The peak at δ = 4.75 corresponds to the hydroxy protons resonance of the branching unit in PTHF_OH_ (Figure 2b), the one at δ = 0.87 ppm can be attributed to the methyl group protons resonance of the branching unit in PTHF_but_ (Figure 2c), and the one at δ = 2.48 ppm can be associated to the methylene protons resonance of the branching unit in PTHF_acid_ (Figure 2d) appear respectively, suggesting all the double bonds in UPTHF modified via thiol-ene reaction. Moreover, Figure 3 shows the FTIR spectra of UPTHF, PTHF, and the following PTHF derivatives: PTHF_OH_, PTHF_but_, and PTHF_acid_. The appearance of a stretching vibration peak at 3340 cm^−1^ (characteristic absorption peaks of the hydroxyl group in PTHF_OH_), a spike vibration peak at 1458 cm^−1^ (characteristic absorption peaks of the methyl group in PTHF_but_), and a stretching vibration peak at 1705 cm^−1^ (characteristic absorption peaks of carboxyl group in PTHF_acid_) also confirms the complete consumption of the double bonds and successful introduction of branch units. Furthermore, the SEC traces of PTHF_OH_, PTHF_but_, and PTHF_acid_ are monomodal, and all the elution parks were located at the higher molecular weight side, similarly to the SEC traces of UPTHF (Figure 4). The *M*_n_,_SEC_ values of the PTHF_OH_, PTHF_but_, and PTHF_acid_ were 6500, 7900 and 9300 g mol^−1^. Besides, the *M*_w_/*M*_n_ of PTHF_OH_, PTHF_but_, and PTHF_acid_ were 1.46, 1.48, and 1.36, respectively. Therefore, based on the above spectral analyses and SEC measurements, we infer that a set of branch units can be efficiently grafted to the backbone of UPTHF via the blue-light photocatalytic thiol-ene reaction.

Although the side-chain functionalized PTHFs have a backbone structure similar with PTHF, the presence of different side-groups in the PTHFs can lead to chemical physical properties which are different to those exhibited by PTHF. Different performance and further application of the side-chain functionalized PTHFs were systematically studied as follows.

### 3.3. Thermal Properties of PTHF_OH_ and PTHF_but_

**Figure 5:**
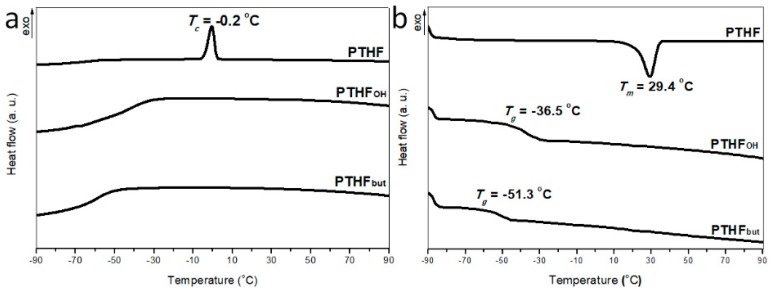
Differential scanning calorimeter (DSC) cooling scans (**a**) and heating scans (**b**) at 5 °C min^−1^ after melting at 90 °C for the indicated polymers.

It is well established that PTHF can be used to synthesize polyurethane materials. However, due to its crystallization, the application of PTHF has been limited to low temperatures. Theoretically, the addition of an alcohol group and butyl branching units could drastically change the thermal properties. [36] To assess the thermal properties and glass transition temperature, the melting and crystallization behaviors of PTHF (*M*_n_ = 3000 g mol^−1^, having a molecular weight similar to the main chains of the PTHF derivatives), PTHF_OH_, and PTHF_but_ were investigated via DSC. As it can be seen from Figure 5, PTHF is crystalline and exhibits a fine single peak during the crystallization (*T_c_* = −0.2 °C) and melting (*T_m_* = 29.4 °C) processes. As we expected, compared with PTHF, the PTHF_OH_ and PTHF_but_ were completely amorphous, with glass transition temperatures at −36.5 and −51.3 °C, respectively. Meanwhile, various brunching units could lead to applications in different fields. The PTHF_OH_ abundance of active hydroxyl-side groups can be used as a hydrogel material, whereas PTHF_but_ being completely amorphous at low temperatures, can be applied as polyurethanes and polyetheresters in very cold areas.

### 3.4. pH-Responsive Behavior of PTHF_acid_

**Figure 6:**
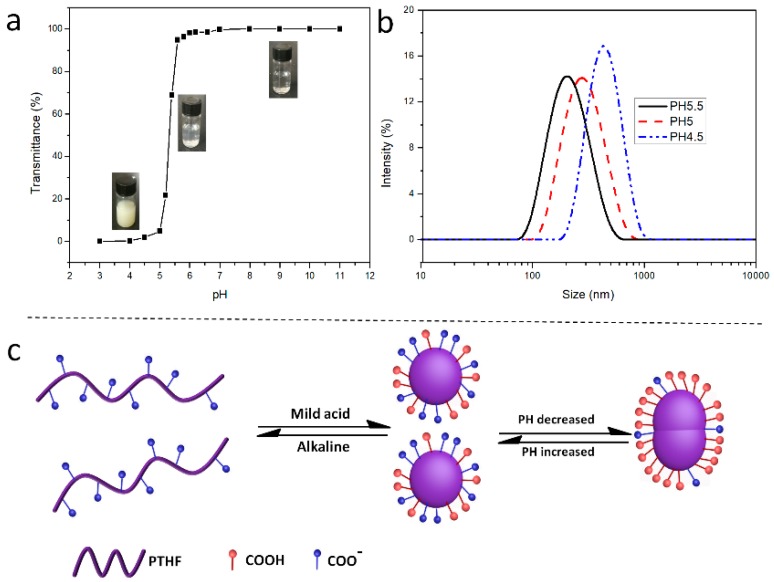
Transmittances of PTHF_acid_ solutions with different pH values (**a**). Dynamic light scattering (DLS) traces of aggregates formed by the self-assembly of PTHF_acid_ in aqueous solution at pH 6, 5, and 4 (**b**), [Polymer] = 0.5 mg mL^−1^. Schematic illustration of the mechanism for the pH-triggered dissolution- assembly-disassembly transition behavior (**c**).

**Figure 7:**
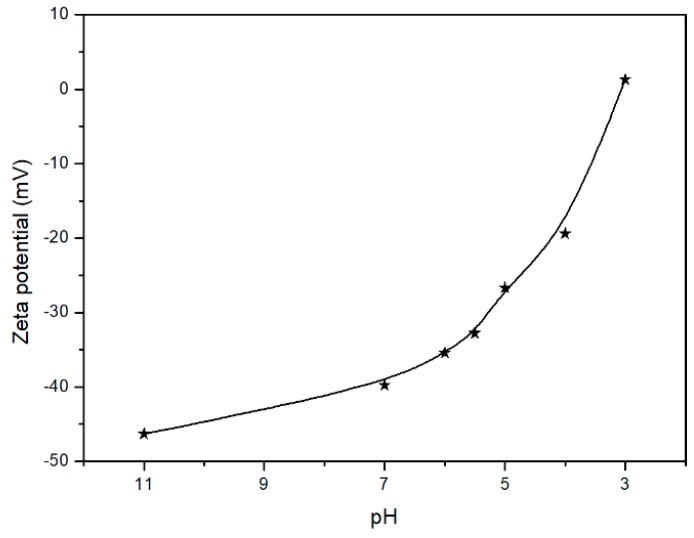
Zeta potential of PTHF_acid_ solution at varying pH.

Recently, the interest for pH-responsive polymers has remarkably increased. PTHF_acid_ composed with a hydrophobic PTHF backbone and hydrophilic carboxyl side groups is a promising pH-responsive polymer, which can self-assemble into aggregates in aqueous solution at appropriate conditions. In general, pH-responsive polymers that dissolve in aqueous solution can show dissolution-assembly-aggregation peculiarity with the decrease of pH. This transformation process can be observed macroscopically via UV-Vis spectroscopy. As shown in Figure 6a, the color of the polymer solution changes from completely transparent to pale blue opalescent and then, to fully opalescent, when the pH decreased from 9.0 to 5.0 to 3.0. The UV transmittance of the polymer solution gradually moved from 100% to 20% and finally to 0%, following a pH decreasing from 11 to 3, thus proving the transformation process of pH-responsive polymers (shown in Figure 6a). Moreover, the size and distribution of the PTHF _acid_ aggregates in aqueous solution with pH 6.0, 5.0, and 4.0 were estimated via DLS. The Z-averaged diameters (D_z_) of the PTHF_acid_ aggregates were 125, 274, and 447 nm, when the pH values were 6.0, 5.0, and 4.0, respectively (Figure 6b); the size distributions presented a narrow polydispersity at 0.26, 0.18, and 0.11, respectively, further indicating that the transformation process of PTHF_acid_ in aqueous solution follows the dissolution-assembly-aggregation transition behavior as pH decreases. The mechanism of the abovementioned pH-triggered dissolution-assembly-aggregation transition behavior is depicted in Figure 6c. Two types of the carboxyl group can be identified in different conditions: The ionized and non-ionized carboxyl ones. In an alkaline environment, carboxyl groups are all ionized, and PTHF_acid_ dissolves in aqueous solution as monomers (dissolution). In mild acidic conditions, ionized and non-ionized carboxyl groups coexist, and PTHF_acid_ exists as an amphiphilic homopolymer forming solid spherical micelles (assembly). With the decrease of the pH, the amount of non-ionized carboxyl groups increases, and PTHF_acid_ is more hydrophobic, enabling the micelles to aggregate and to form larger micelles (aggregation). As shown in Figure 7, the ζ-potential absolute value decreases sharply as pH value decreases, confirming the validity of the mechanism of the pH-triggered dissolution-assembly-aggregation transition behavior. 

### 3.5. Backbone-Thermoresponsive Behavior of PTHF_mPEG_

**Figure 8:**
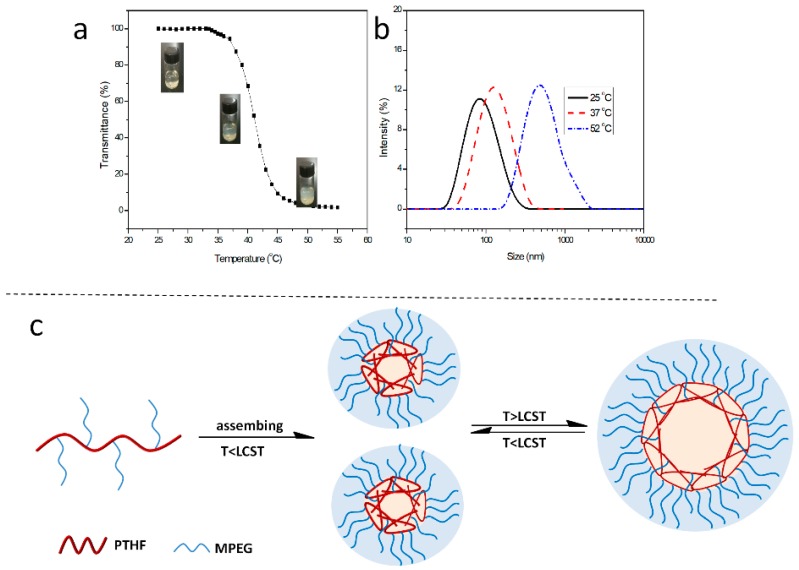
Transmittances of PTHF_mPEG_ solution in different temperature (**a**). DLS traces of aggregates formed by the self-assembly PTHF_mPEG_ in aqueous solution at temperature 25, 37 and 52 °C (**b**), [Polymer] = 0.5 mg mL^−1^. Schematic illustration of the mechanism for the temperature-triggered self-assembly behavior (**c**).

In the past decades, the backbone-thermoresponsive polymers have been extensively studied because of their various promising applications, especially in the synthesis of hyperbranched polymers, which are indeed prepared by combining both hydrophobic and hydrophilic components into the polymer backbone to form a hydrophobic-hydrophilic balance system [37,38,39]. However, up to now, the brush polyether with a backbone-thermoresponsive has not yet been synthesized. Here, we employed the same thiol-ene reaction between UPTHF and mPEG-SH to synthesize PTHF_mPEG_. When all the double bonds of UPTHF have reacted with mPEG-SH, the obtained polymer (i.e., PTHF_mPEG1_) can only self-assemble into micelles without exhibiting any thermoresponsive properties in aqueous solution (as determined by the analysis of the ^1^H NMR spectrum of PTHF_mPEG1_ in Figure S4a). The possible reason is that the content of mPEG is too rich in hydrophilic components. Fortunately, by regulating the number of hydrophilic branched chains of mPEG to form a hydrophobic-hydrophilic balance system with the backbone of PTHF having ~5.3 branch units of mPEG, we could successfully prepare the backbone-thermoresponsive brush polyether (i.e., PTHF_mPEG2_). The ^1^H NMR spectrum of PTHF_mPEG2_ is displayed in Figure S4b. UV-Vis spectroscopy was used to determine lower critical solution temperature (LCST) of the backbone-thermoresponsive brush polyether. The LCST of PTHF_mPEG2_ was determined to be 35.2 °C (Figure 8a). Meanwhile, the sizes and distribution of the PTHF_mPEG2_ aggregates in aqueous solution at 25, 37 and 52 °C were assessed via DLS. When the temperature had been set at 25 °C (i.e., below LCST), the D_z_ and the size distributions of the PTHF_mPEG2_ were 91 nm and 0.26, respectively. In contrast, when the temperatures had set at 37 and 52 °C (i.e., above LCST), the D_z_ of the PTHF_mPEG2_ gradually increased to 116 and 451 nm, respectively, whereas the size distributions gradually decreased to 0.16 at 37 °C and 0.14 at 52 °C (Figure 8b). The simple mechanisms of the thermoresponsive aggregation of the PTHF_mPEG2_ are shown in Figure 8c. The PTHF_mPEG2_ self-assembles into the stabilized nano-micelles below the LCST. When the temperature is above LCST, the hydrophilic-hydrophobic balance breaks and the micelles gradually aggregate into the larger micelles.

### 3.6. In Vitro Cytotoxicity of PTHF_acid_ and PTHF_mPEG_

**Figure 9:**
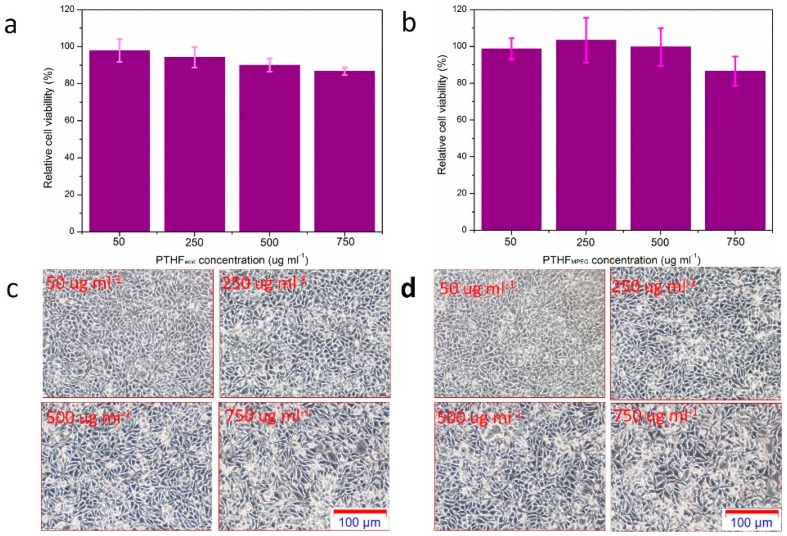
The cell viability of A549 cells after incubation with PTHF_acid_ and PTHF_mPEG_ for 24 h (PTHF_acid_ for (**a**) and PTHF_mPEG_ for (**b**), optical microscopy images of A549 cells after incubation with different concentrations of PTHF_acid_ and PTHF_mPEG_ for 24 h (PTHF_acid_ for (**c**) and PTHF_mPEG_ for (**d**)).

pH-responsive and thermo-responsive polymers are widely used in biomaterials, especially in drug delivery. Therefore, the biocompatibility is of great importance. We, thus, evaluated the in vitro cytotoxicity of micelles of PTHF_acid_ and PTHF_mPEG_ in A549 cells by performing the CCK-8 assay to ensure the potential application for biomaterials. As shown in Figure 9a,b, the cell viabilities of A549 cells after incubation for 24 h were both more than 85%, even at a high concentration of 750 μg mL^−1^. Furthermore, the optical microscopy observation (Figure 9c,d) of A549 cells treated with PTHF_acid_ and PTHF_mPEG_ of different concentrations show almost no dead cells, implying good biocompatibility with living cells. 

## 4. Conclusions

Herein, we reported the synthesis of side-chain functionalized PTHFs. The blue-light photocatalytic thiol-ene “click” reaction was employed to prepare a series of side-chain functionalized PTHFs containing hydroxyl, butyl, and carboxylate groups, as well as PEG branch units linking in the PTHF backbone. Distinct branch units in the PTHF derivatives led to different performances. For instance, PTHF_OH_ and PTHF_but_ were all completely amorphous; PTHF_acid_ and PTHF_mPEG_ were pH-responsive and thermo-responsive polymers, respectively, both exhibiting good biocompatibility with living cells. Therefore, the blue-light photocatalytic thiol-ene “click” reaction represents a versatile strategy to synthesize multiple side-chain functionalized PTHFs with designed properties.

## Figures and Tables

**Table 1:** Synthesis of UPTHF with different polymerization technology.

Sample	Solvent	Temperature (°C)	Catalyst	Reaction Time (h)	*M*_n_ (g mol^−1^)	*M*_w_/*M*_n_
1	acetone	reflux	NaH	48	423	1.31
2	acetone	reflux	KOH	72	4306	1.48
3	acetone	reflux	KOH	48	1806	1.72
4	acetone	reflux	K_2_CO_3_	72	279	1.18

## Supplementary Materials

The following are available online at https://www.mdpi.com/2073-4360/11/4/583/s1, Figure S1: ^1^H NMR spectrum of UPTHF (*M_n_* = 4300 g mol^−1^) in CDCl_3_; Figure S2: ^13^C NMR spectrum of UPTHF (*M*_n_ = 4300 g mol^−1^) in CDCl_3_; Figure S3: ^1^H NMR spectrum of PTHF_acid_ in DMSO-*d*_6_; Figure S4: ^1^H NMR spectra of PTHF_mPEG1_ (a) and PTHF_mPEG2_ (b) in DMSO-*d*6.
