# Supplementary material for: Synthesis and Properties of Side-Chain Functionalized Polytetrahydrofuran Derivatives via the Blue-Light Photocatalytic Thiol-Ene Reaction
Source: Polymers (Basel). 2019 Apr 1;11(4):583. doi: 10.3390/polym11040583 (PMC6523133; doi:10.3390/polym11040583)
Supplement: Supplementary file 1 [file polymers-11-00583-s001.pdf]

SUPPORTING INFORMATION

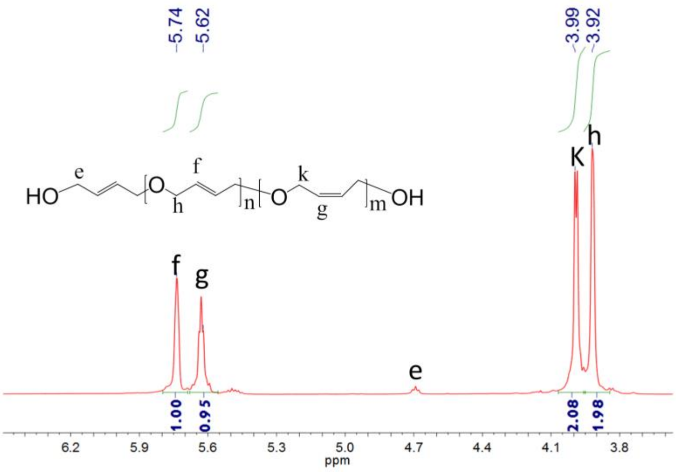

Figure S1. ^{1}H NMR spectrum of UPTHF (M_n = 4300 g mol^{-1}) in $CDCl_3$

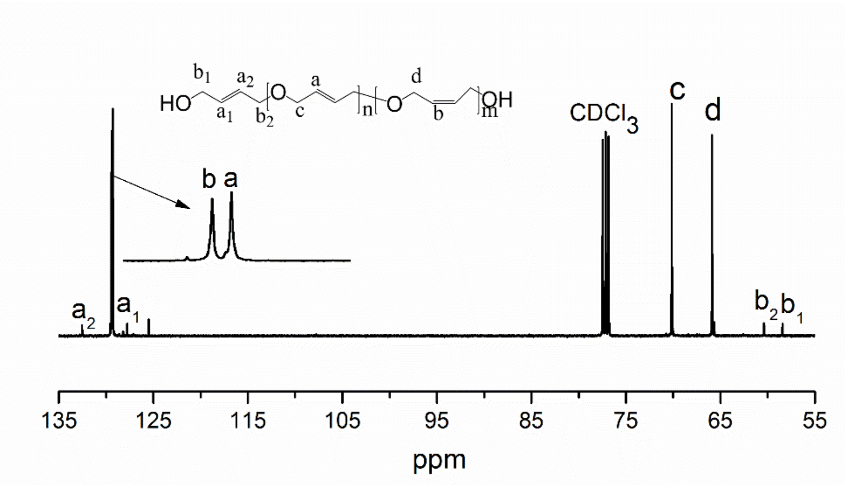

Figure S2. ^{13}C NMR spectrum of UPTHF (M_n = 4300 g mol^{-1}) in $CDCl_3$

Synthesis of PTHF derivatives (PTHF$_{acid}$) via UV light photocatalytic thiol-ene reaction

A 20 mL quartz tube loaded with a stir bar, PBD (260 mg; 0.2 mmol, -C=C-, 3.7 mmol), 3-mercaptopropionic acid (3.95 mL, 45 mmol), and DMAP (7.0 mg, 0.026 mmol) were dissolved in solution of THF (4mL) and irradiated by a UV light (365nm) for 24 h at room temperature. After reaction, unreacted 3-mercaptopropionic and THF were isolated by dialyzed (molecular weight cut off: 300) against distilled water for 48 h, and final product was obtained by freeze drying.

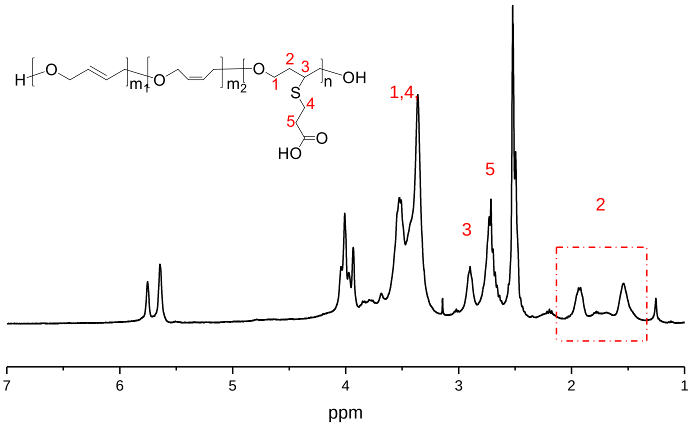

Figure S3. ^{1}H NMR spectrum of $PTHF_{acid}$ in DMSO-d_6 .

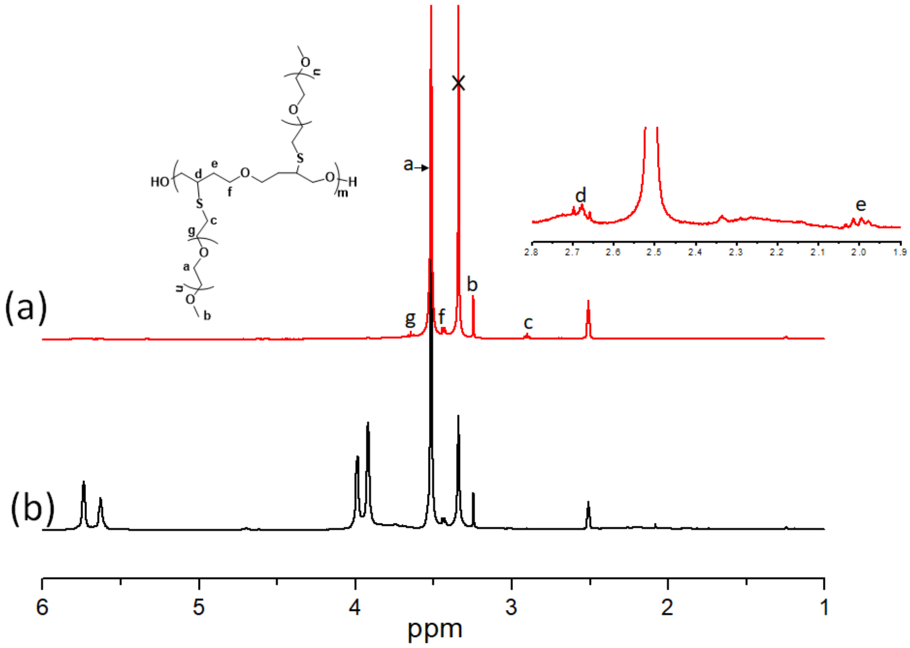

Figure S4. ^{1}H NMR spectra of $PTHF_{mPEG1}$ (a) and $PTHF_{mPEG2}$ (b) in $DMSO_{\text{-d6}}$
